# Influence of Activator Modulus and Water-to-Binder Ratio on Mechanical Properties and Damage Mechanisms of Lithium-Slag-Based Geopolymers

**DOI:** 10.3390/ma18204695

**Published:** 2025-10-13

**Authors:** Shujuan Zhang, Chiyuan Che, Haijun Jiang, Ruiguo Zhang, Yang Liu, Shengqiang Yang, Ning Zhang

**Affiliations:** 1 School of Safety Engineering, China University of Mining and Technology, Xuzhou 221116, China; 2 School of Civil Engineering, Xuzhou University of Technology, Xuzhou 221018, China; 3 State Key Laboratory for Fine Exploration and Intelligent Development of Coal Resources, China University of Mining and Technology, Xuzhou 221116, China; 4 School of Resources and Environmental Engineering, Inner Mongolia University of Technology, Hohhot 010051, China; 5 Taian Quality Technical Inspection and Testing Institute (Taian Special Equipment Inspection and Research Institute), Tai’an 271000, China; 6 Ordos Ulan Coal (Group) Co., Ltd., Ordos 017000, China

**Keywords:** geopolymer, modulus of activator, water-to-binder ratio, mechanical property, acoustic emission, failure mechanism

## Abstract

The synergistic preparation of geopolymer from lithium slag, fly ash, and slag for underground construction can facilitate the extensive recycling of lithium slag. The effects of different activator moduli and water–binder ratios on the mechanical properties and damage mechanisms of the lithium-slag-based geopolymer were investigated by uniaxial compression tests and acoustic emission (AE) monitoring. The results show that, based on a comprehensive evaluation of peak stress, crack closure stress, plastic deformation stress, and elastic modulus, the optimal activator modulus is determined to be 1.0, and the optimal water-to-binder ratio is 0.42. At low modulus values (0.8 and 1.0) and low water–binder ratio (0.42), the AE events exhibit a steady pattern, indicating slow crack initiation and propagation within the geopolymer; with the increasing activator modulus and water-to-binder ratios, the frequency of AE events increases significantly, indicating more-frequent crack propagation and stress mutation within the geopolymer. Similarly, when the modulus is 0.8 or 1.0 and the water–binder ratio is 0.42, the sample presents a macroscopic tensile failure mode; as the modulus and water–binder ratio increase, the sample presents a tensile–shear composite failure mode. The energy evolution laws of geopolymer specimens with different activator moduli and water-to-binder ratios were analyzed, and a damage constitutive model was established. The results indicate that, with optimized mix proportions, the material can be used as a supporting material for underground spaces.

## 1. Introduction

As new energy vehicles are rapidly advancing in China, the requirement for lithium batteries has been steadily rising, and the stockpile of lithium slag (LS), a byproduct of lithium salt production, has increased dramatically over the past few years [1,2]. In China, the annual stockpile has exceeded 12 million tons, and the global cumulative stockpile has surpassed 1.2 billion tons. However, the overall utilization rate is only 6%. As a result, increasing attention is being paid to the recycling and utilization of LS [3,4]. In the construction industry, LS as an admixture in cement mixtures or concrete is a key area for large-scale consumption of LS [5,6]. The construction of underground buildings during mining activities is also a key method for the disposal and utilization of industrial solid wastes. For example, the construction of roadside backfill [7,8,9], structural backfill [10,11], and artificial pillars [12,13] can consume a large amount of fly ash (FA) and slag (SG). If LS can be used together with FA, SG, and other materials in the construction of underground buildings, this can potentially facilitate the large-scale recycling of LS and solve the soil and water pollution issues caused by LS accumulation.

With the deepening research on geopolymer, the use of industrial solid wastes with geopolymerization activity has been gradually emphasized for the development of alkali-activated geopolymer materials in recent years [14,15,16]. FA and SG are commonly used active aluminosilicate raw materials for the preparation of geopolymers [17]. Some researchers have proposed the use of LS and other industrial solid wastes to prepare geopolymers. Luo et al. [18] prepared an LS-based geopolymer cement by a one-part mixing method and studied the effect of LS on the processability and mechanical properties of the geopolymer. Javed et al. [19] investigated the dilution effect of adding silica fume and FA as chemical modifiers in an LS-based geopolymer on the sulfate component in the pore solution and analyzed the coagulation behavior and compressive strength of LS-based geopolymer containing silica fume and FA. Shah et al. [1] developed an ambiently cured, eco-friendly single-component geopolymer using LS and SG as aluminum silicate precursors and anhydrous sodium silicate as an alkaline activator, and comprehensively evaluated both the fresh properties and hardened properties of the prepared material. The existing studies [20,21] have reported that the synergistic use of LS and FA, SG can improve the reactivity of LS, and the prepared geopolymer has reliable mechanical properties. Therefore, using LS, FA, and SG to prepare geopolymers as underground building materials will have important significance in engineering applications. However, the mechanical performance requirements for geopolymers in underground structures differ from those in surface structures, mainly due to the influence of mining activities. These include roof collapses, floor heave, and disturbances from the extraction of adjacent coal seams, all of which demand that underground structures possess reliable mechanical properties and damage resistance. Otherwise, damage, cracking, or collapse of underground structures would pose serious threats to the safety of personnel and equipment underground.

The water–binder ratio and the activator modulus are the critical factors in this design process. Water is an important component of the alkaline activation process of aluminosilicate industrial solid waste, and its dynamic transformation during the reaction is one of the important manifestations of the formation of alkaline activation products [22,23,24]. The activator modulus, defined as the molar ratio of SiO_2_ to Na_2_O, is used to indicate the silicon content in the sodium silicate solution and also to evaluate the strength of the activator solution. It controls the silicon source concentration in the solution, thereby affecting the structure and formation rate of geopolymer gels [25]. Therefore, the water–binder ratio and activator modulus are the key factors affecting the mechanical properties and damage resistance of geopolymers. Acoustic emission (AE) technology is frequently employed for the non-destructive testing and evaluation of cement-based materials [26]. Using AE to monitor the deformation and failure process of a geopolymer can evaluate the damage resistance of the geopolymer.

However, the current understanding of the coupled effects of water-to-binder ratio and activator modulus on crack evolution and energy dissipation in lithium-slag-based geopolymers remains limited. In this study, the effects of activator modulus and water–binder ratio on the mechanical properties and damage mechanism of lithium-slag-based geopolymer samples were explored. The stress–strain curve behaviors, elastic modulus, peak strain, peak stress, crack closure stress, and plastic deformation stress of geopolymer under the influence of activator modulus (0.8, 1.0, 1.2, and 1.4) and water–binder ratio (0.42, 0.43, 0.44, and 0.45) were analyzed through uniaxial compression tests. The crack initiation and propagation laws within geopolymer samples were characterized by AE ringing count, *b*-value, and *S*-value. The crack propagation mode of geopolymer samples was analyzed by RA-AF correlation. The influence of activator modulus and water-to-binder ratio on the macroscopic failure modes of geopolymer specimens was discussed. Finally, the energy evolution laws of geopolymer specimens with different activator moduli and water-to-binder ratios were analyzed, and a damage constitutive model for geopolymer specimens was established. This research is of great significance for understanding the failure behavior of a lithium-slag-based geopolymer under different activator moduli and water–binder ratio conditions, and ensuring its reliable application in engineering practices.

## 2. Materials and Methods

### 2.1. Raw Materials and Sample Preparation

#### 2.1.1. Raw Materials

In the geopolymer fabrication process, LS, FA, and SG were selected as active aluminosilicate raw materials; municipal tap water was used as mixed water; and flaky NaOH combined with liquid sodium silicate was used as an activator. The LS was prepared by spodumene acid roasting method. After LS was extracted, it was ground by a ball mill for 10 min and then calcined at 700 °C for 2 h to improve the reactivity of LS [6]. Measured by Rigaku Ultima IV X-ray diffractometry (Rigaku Corporation, Tokyo, Japan), the main phases of LS were spodumene (HALSi_2_O_6_) after delithiation, gypsum (CaSO_4_·2H_2_O), and quartz (SiO_2_); the main mineral components of FA were quartz and hematite, and SG exhibited an amorphous structure with no detectable crystalline phases. The average particle sizes (D50) of LS, FA, and SG measured by the Malvern Mastersizer 2000 laser particle-size analyzer (Malvern Instruments Ltd., Malvern, UK) were 26.16 μm, 25.35 μm, and 12.39 μm, respectively. Table 1 shows the chemical compositions of LS, FA, and SG measured by the X-ray fluorescence spectrometer of Thermo Fisher Scientific ARL Perform’X (Thermo Fisher Scientific, Waltham, MA, USA). The initial modulus of the liquid sodium silicate used was 2.31, the content was 42%, and the Baume degree was 50. The concentration of flaky sodium hydroxide was 97%.

#### 2.1.2. Sample Preparation

The LS, FA, and SG dosages were fixed at 9%, 41%, and 50%, and the activator dosage was 10% (the mass of the activator accounted for that of the active aluminosilicate raw material). The activator solution was prepared using sodium silicate, sodium hydroxide, and water. According to Lv et al. [27], the optimal molar ratio of sodium silicate ranges from 0.8 to 1.2. When the molar ratio of sodium silicate is too high, the solution becomes excessively viscous, which hinders the continuation of the reaction; conversely, when the molar ratio is too low, the ion concentration in the solution becomes insufficient, resulting in a weakened activation effect. The activator modulus was set to 0.8, 1.0, 1.2, and 1.4, and the activator modulus was adjusted by adding NaOH to liquid sodium silicate [27]. Based on research results obtained from our tests, the water–binder ratio was set to 0.42, 0.43, 0.44, and 0.45, respectively. This ratio guaranteed that the slump of the slurry exceeded 200 mm, thereby satisfying the fluidity requirements for the slurry in underground filling projects (slump > 152 mm) [28]. Table 2 shows the detailed mixing ratios.

**Figure 1:**
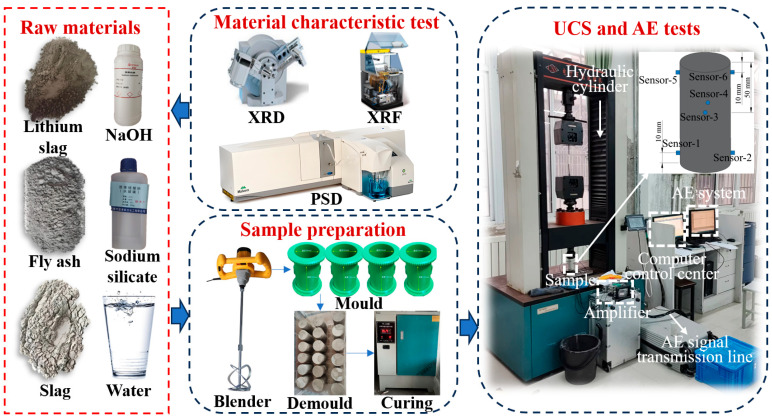
Test process.

Before preparing geopolymer samples, the activator was dissolved in water for 3 min and stirred evenly, then allowed to cool naturally. After LS, FA, and SG were mixed and stirred for 1 min, the solution of the activator and water was poured in and stirred for 3 min [18]. The mixture was then poured into a cylindrical mold with a diameter of 50 mm and a height of 100 mm. After standing at room temperature for 12 h, the samples were demolded and placed in a YH-40B standard constant-temperature and -humidity curing box with a curing temperature of (20 ± 2) °C and a relative humidity of (95 ± 2)%. The curing age was set as 28 days [29]. Three samples were prepared for each mixing proportion. Figure 1 shows the sample preparation process.

### 2.2. Experimental Equipment

The CSS electronic universal testing machine (produced by Changchun Testing Machine Research Institute, Changchun, China) was used to perform uniaxial compression tests on the samples in line with the ASTM C39 standard [30]. The loading mode of the electronic universal testing machine was displacement control, and the loading rate was 0.5 mm/min. AE monitoring was performed simultaneously with uniaxial compression testing. The PCI-2 monitoring system of the American Physical Acoustics Company was used as an AE monitoring system, with 6 sensors. Figure 1 shows the sensor layout of the AE monitoring system. The operating frequency of the sensor was 125–750 kHz, the resonant frequency was 140 kHz. To minimize the impact of environmental noise, the acquisition threshold was set to 40 dB, the gain to 40 dB, and the sampling frequency to 2 MHz [31]. Figure 1 shows the entire experimental process.

## 3. Results and Discussion

### 3.1. Mechanical Properties

#### 3.1.1. Stress–Strain Curves, Elastic Modulus, and Peak Strain

**Figure 2:**
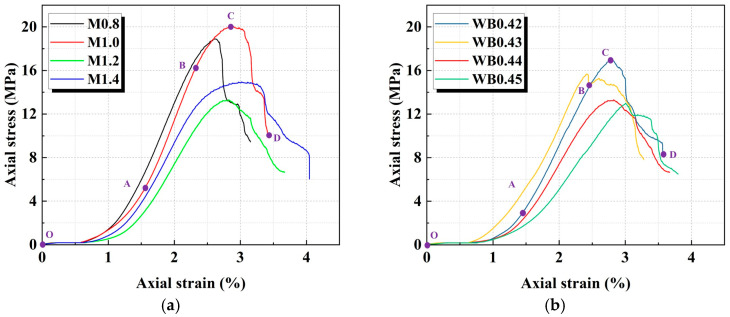
Stress–strain curves of geopolymer sample: (**a**) different activator moduli; (**b**) different water–binder ratios.

Figure 2 displays the stress–strain curves of geopolymer samples with different activator moduli and water–cement ratios. According to the studies by Che et al. [11], the stress–strain curve can be divided into four stages: pore compaction, linear elasticity, plastic yielding, and post-peak failure. In the pore compaction stage (OA), due to the existence of the original open structural surface, pores and microcracks within the geopolymer samples, the stress increases slowly with the increase in strain during initial loading. As a result, the stress–strain curve exhibits a concave shape. This occurs as the external axial load leads to a gradual closure of the sample’s internal pore structure, and then the geopolymer sample exhibits significant nonlinear compression characteristics [28]. With the gradual closure of the pores, the geopolymer sample enters the linear elastic stage (AB). At this stage, the stress–strain relationship becomes increasingly linear. In addition, the stress inside the sample is uniformly transmitted, and no obvious crack propagation is formed. As the stress increases further, the geopolymer sample enters the plastic yield stage (BC). At this time, the stress growth rate slows down and the stress–strain curve shows an upward convex trend, indicating that the material transitions from elastic deformation to plastic yield [31]. In the post-peak stage (CD), after the geopolymer sample reaches its ultimate bearing capacity, the stress drops rapidly, and the curve shows a steep decline trend. The macro cracks have penetrated through the geopolymer sample, causing the material failure.

**Figure 3:**
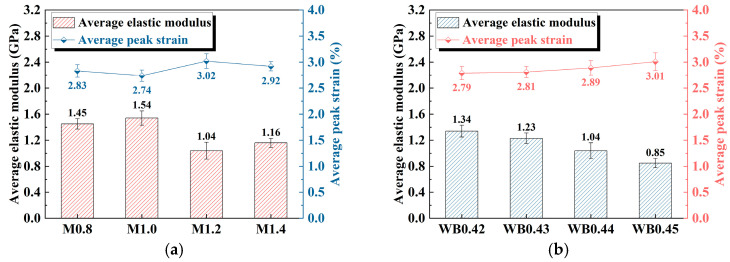
Elastic modulus and peak strain of geopolymer samples: (**a**) different activator modulus; (**b**) different water–binder ratios.

Figure 3 reveals the elastic modulus and peak strain of geopolymer samples under different activator moduli and water–binder ratios. As shown in Figure 3a, with the increase in the activator modulus, the elastic modulus of the geopolymer sample experiences an increase–decrease–increase trend. When the activator modulus is 1.0, the elastic modulus reaches the maximum value of 1.54 GPa; when the activator modulus is 1.2, the elastic modulus reaches the minimum value of 1.04 GPa, which is 32.47% lower than that at the activator modulus of 1.0. Generally, high-modulus activators are less active than low-modulus activators [32], which in turn reduces the degree of reaction of the active aluminosilicate raw materials. Therefore, when the activator modulus reaches 1.2 and 1.4, the elastic modulus decreases. An increase in the activator modulus indicates a higher relative content of Na_2_O and a reduced content of SiO_2_, resulting in an insufficient supply of silicon sources in the solution [27]. As the silicon source is a key element in the formation of the geopolymer gel network, a low activator modulus leads to inadequate participation of SiO_2_ in the reaction. Consequently, the resulting gel structure becomes less dense and continuous, with a reduced degree of crosslinking [33]. This causes a loose microstructure of the material, thereby reducing the overall stiffness of the geopolymer, which is reflected in a lower elastic modulus.

The peak strain shows a decrease–increase–decrease pattern with the increase in activator modulus. When the activator modulus is 1.2, the peak strain reaches its maximum value at 3.02%; when the activator modulus is 1.0, the peak strain decreases to its minimum value of 2.74%. Compared with the activator modulus of 1.2, the peak strain is reduced by 9.27%.

As shown in Figure 3b, with the increase in the water–binder ratio, the elastic modulus gradually decreases, while the peak strain shows a corresponding increasing trend. This is mainly due to the increase in porosity and decrease in matrix density caused by the increase in the water–binder ratio [34]. When the water–binder ratio is lower, the water content in the slurry is relatively small, and the gel formation is more complete, leading to a denser internal structure of geopolymer. Consequently, the material exhibits a higher elastic modulus and stronger deformation resistance. A higher water–binder ratio makes the micro-structure of the material looser, and under the action of local stress concentration, the geopolymer sample undergoes greater deformation before reaching the ultimate bearing capacity.

Based on the above analysis, it can be concluded that the elastic modulus, peak stress, and peak strain of lithium-slag-based geopolymers are significantly lower than those of concrete- and conventional-slag-based geopolymers. The main reasons are the low reactivity of lithium slag and its high content of inert crystalline phases (such as quartz and lithium-extracted spodumene), which hinder the full progression of the geopolymerization reaction. This results in a gel structure that is insufficiently dense and continuous, thereby reducing the overall mechanical properties of the material. However, the strength requirement for underground support materials is generally below 10 MPa; thus, the materials prepared in this study can be used as supporting materials for underground spaces.

#### 3.1.2. Peak, Crack Closure, and Plastic Deformation Stress

**Figure 4:**
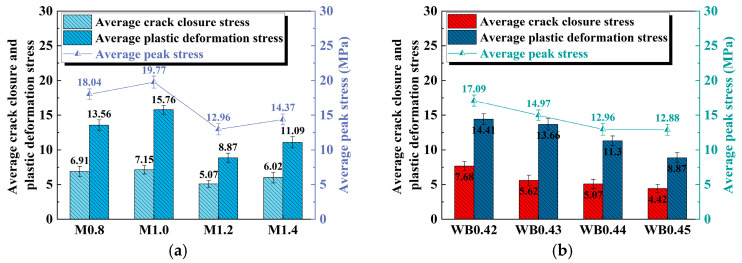
Peak stress, crack closure stress, and plastic deformation stress of geopolymer samples: (**a**) different activator moduli; (**b**) different water–binder ratios.

The stresses at the starting point and end point of the linear elastic stage are defined as crack closure stress and plastic deformation stress to characterize the elastic–plastic stress threshold of the sample [35]. Figure 4 illustrates the peak stress, crack closure stress, and plastic deformation stress of geopolymer samples under the influence of different activator moduli and water–cement ratios.

As displayed in Figure 4a, the peak stress first increases, then decreases, and finally increases as the modulus of the activator increases. It reaches the maximum value (19.77 MPa) when the modulus is 1.0, and then drops to the minimum (12.96 MPa) when the modulus is 1.2, which is a decrease of 34.35% compared to that at the modulus of 1.0. The changing trends in crack closure stress and plastic deformation stress are consistent with those of peak stress. This shows that the activator modulus not only affects the ultimate load-bearing capacity of geopolymer samples, but also directly regulates the elastic–plastic properties.

As displayed in Figure 4b, as the water–binder ratio increases from 0.42 to 0.45, the peak stress, crack closure stress, and plastic deformation stress all show a gradual downward trend. The higher crack closure stress indicates that the material can effectively resist crack initiation and propagation during the initial loading stage, while the higher plastic deformation stress reflects that the material has a strong deformation coordination ability, so that it can still maintain good stability before reaching the peak stress. However, as the water–binder ratio increases, the free water content in the slurry increases, resulting in an increase in the pores formed after solidification and hardening. Cracks are more likely to initiate and expand, which is manifested as a decrease in crack closure stress, plastic deformation stress, and peak stress.

### 3.2. AE Ringing Counts

**Figure 5:**
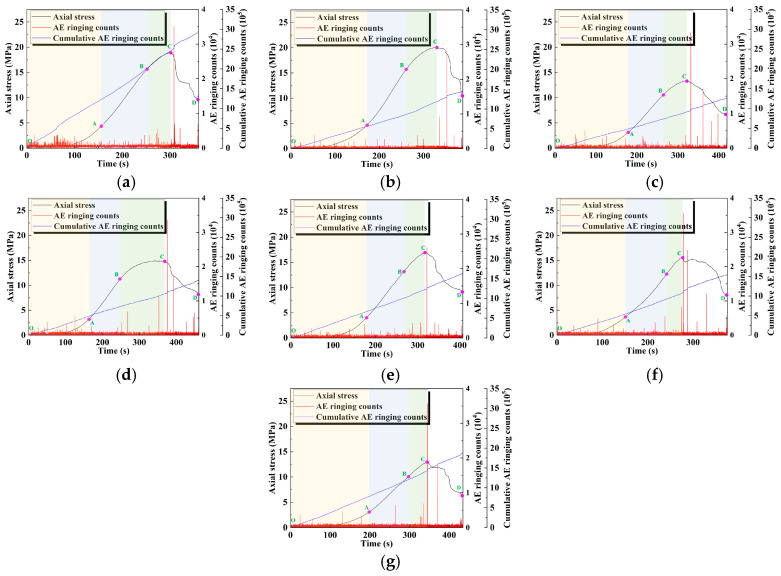
Evolution of AE ringing counts with time–stress curves: (**a**) M0.8; (**b**) M1.0; (**c**) M1.2/WB0.44; (**d**) M1.4; (**e**) WB0.42; (**f**) WB0.43; (**g**) WB0.45.

The AE ringing counts can reflect the degree of damage and degradation of the internal structure of the material during loading [36]. Figure 5 shows the evolution of the AE ringing count of geopolymer samples with time–stress under the influence of different activator moduli and water–binder ratios. In general, ringing counts in AE events are more active in the pore compaction stage, which is due to the friction of the matrix during the pore closure process; it has the lowest activity in the linear elastic stage, at which the sample is gradually compacted and its internal structure tends to be stable. In the plastic yield stage, microcracks are formed, leading to the gradually increased activity of the AE ringing count. In the post-peak failure stage, the cracks expand rapidly, and the activity of the AE ringing count reaches its peak.

In addition, when the modulus is low (0.8 and 1.0), the AE ringing count is relatively stable, reflecting the uniform stress transfer and the slower generation and propagation of cracks within the geopolymer sample. With the increase in the modulus (1.2 and 1.4), the AE ringing count of the geopolymer sample increases significantly. This indicates that the microcrack propagation and stress concentration inside the geopolymer sample are more significant, resulting in more frequent crack propagation and stress mutation. This trend is also reflected by the significant increase in the cumulative AE ringing counts.

When the water–binder ratio is lower (0.42), the AE ringing count is lower and mainly concentrated in the post-peak failure stage, and the cumulative AE count grows slowly. This indicates that crack propagation is inhibited to a certain extent. When the water–binder ratio is higher (0.43–0.45), the AE activity is significantly enhanced, the crack propagation occurs earlier, and the cumulative AE count grows steeply. This indicates that the higher water–binder ratio aggravates the generation and propagation of microcracks inside the material.

### 3.3. b-Value and S-Value

The generation and propagation of microcracks inside the material will release energy in the form of sound waves and produce AE events. Therefore, AE events can be identified as a microseismic activity, and the deformation and failure scale characteristics of the material can be characterized by the *b*-value, a parameter related to earthquake magnitude and frequency. The Gutenberg–Richter (G-R) relationship is as follows [37]:

(1) lgN=a−bM

where *N* is the number of earthquakes within the range of *M* + Δ*M*; *a* is the constant of seismic activity; *b* is the constant representing the ratio of the number of large and small earthquakes; and *M* is the magnitude of the earthquake, and is generally replaced by the AE amplitude divided by 20.

In this study, the least-squares method was used to calculate the *b*-value of geopolymer samples in AE events. To prevent significant error in the *b*-value calculation caused by a limited number of AE events in a certain period, 200 AE events were set as a calculation period, and the sliding window step was set as 150 AE events. The increase in the *b*-value indicates that the proportion of small-scale damage events increases, and the decrease in the *b*-value indicates that the proportion of large-scale damage events increases. The relatively constant *b*-value represents the balanced distribution of damage events of different scales. The fluctuation in the *b*-value in a small range represents the gradual and stable propagation process of cracks; the fluctuation in *b*-value in a large range represents the sudden and large-scale propagation process of cracks.

The *S*-value can reflect the concentration of the AE source, which is calculated by frequency and amplitude. It can be analyzed together with the *b*-value to infer the propagation degree of cracks. The calculation method of *S*-value is as follows [38]:

(2) $$S=0.117\mathrm{lg}(N+1)+0.029\mathrm{lg}\frac{1}{N}\sum_{i=1}^{N}10^{0.075m_{\mathrm{si}}}+0.00075m_{s}$$

where *m*_si_ represents the amplitude of each AE event; and *m*_s_ represents the maximum amplitude of the AE event.

**Figure 6:**
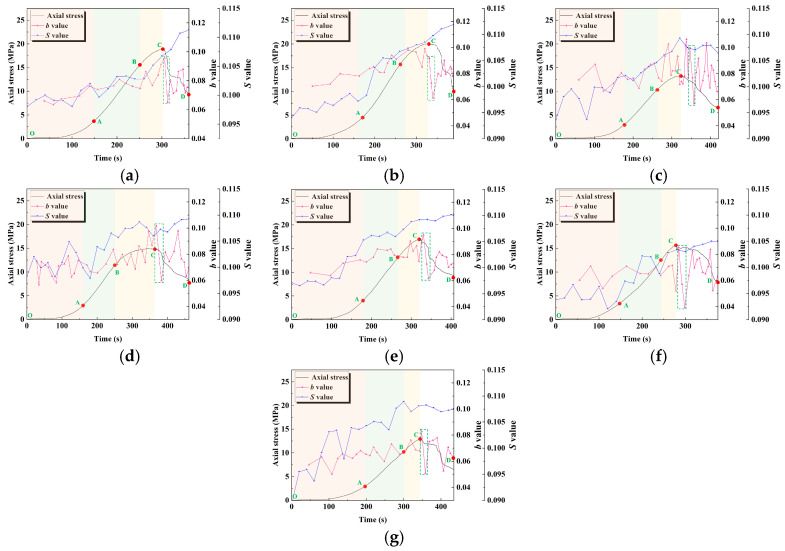
Evolution of *b*-value and *S*-value with time–stress curves: (**a**) M0.8; (**b**) M1.0; (**c**) M1.2/WB0.44; (**d**) M1.4; (**e**) WB0.42; (**f**) WB0.43; (**g**) WB0.45.

Figure 6 shows the evolution of the *b*-value and *S*-value of geopolymer samples with the time–stress curve under the influence of different activator moduli and water–binder ratios. It can be seen that at a lower modulus (0.8 and 1.0), in the pore compaction and linear elastic stages, the *b*-value is relatively stable. This reflects that the crack propagation is relatively uniform and dominated by small-scale damage events, the crack propagation process is gradual, and the AE activity is mainly driven by the gradual accumulation of microcracks. As the modulus increases to 1.2 and 1.4, the fluctuation amplitude of the *b*-value gradually increases and reaches the maximum in the post-peak damage stage (within the green dotted box); this indicates that the crack propagation at this time changes from a gradual mode to a sudden mode, the large-scale damage events inside the geopolymer sample increase, and the crack propagation rate accelerates. In addition, the increase in *S*-value indicates that the concentration of the AE source is enhanced, which further confirms the local energy concentration effect in the crack propagation process. This indicates that the crack propagation is no longer uniform, but has caused strong damage in some local areas.

In addition, at a low water–cement ratio (0.42), the *b*-value fluctuates less, indicating that small-scale damage events dominate, and the crack propagation is relatively stable. As the water–cement ratio increases (0.43 to 0.45), the fluctuation amplitude of the *b*-value increases. This indicates that at this time, the number of large-scale damage events in the crack propagation process gradually increases, and the damage process becomes more severe. The change in *S* value is consistent with the fluctuation trend of the *b*-value, that is, when the water–binder ratio is lower, the *S*-value remains stable, indicating that the AE source is more dispersed and the local energy concentration effect is weak. When the water–binder ratio is higher, *S* value fluctuates significantly, indicating that the local energy concentration phenomenon is more significant during crack propagation, and the localization phenomenon of crack propagation is enhanced.

### 3.4. Crack Propagation Mode

When tensile microcracks occur, the time for the AE waveform to reach the maximum value is short, and the waveform rise angle is large. When shear microcracks occur, the time for the AE waveform to reach the maximum value is long, and the waveform rise angle is small. Therefore, the microcrack propagation mode of the geopolymer sample can be analyzed from a statistical perspective through *RA* (reflecting the rise angle) and *AF* (average frequency). The calculation functions of *RA* and *AF* are as follows [37]:

(3) $$RA=\frac{RT}{A}$$

(4) $$AF=\frac{C}{DT}$$

where *RT* is the rise time of the AE signal wave; *A* is the maximum amplitude; *C* is the number of AE ringing counts; and *DT* is the duration.

**Figure 7:**
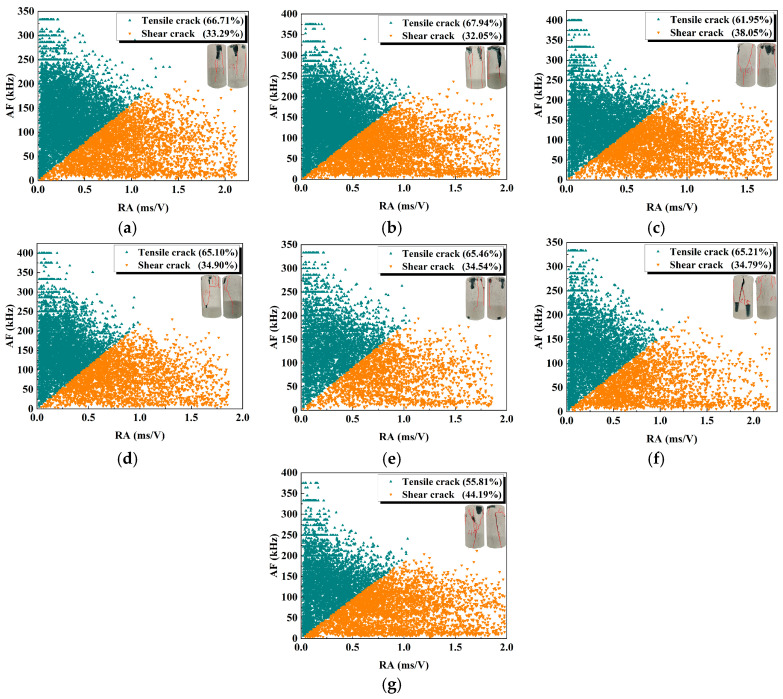
*RA*-*AF* relationship and statistical results of tensile–shear cracks: (**a**) M0.8; (**b**) M1.0; (**c**) M1.2/WB0.44; (**d**) M1.4; (**e**) WB0.42; (**f**) WB0.43; (**g**) WB0.45.

Referring to the previous study conducted by Yue et al. [39], the coefficient *k* is used as the classification threshold of *AF* and *RA*; that is, when *AF*/*RA* is less than *k*, the crack is defined as a shear crack; when *AF*/*RA* is greater than *k*, it is defined as a tensile crack; *k* is defined as the ratio of the maximum *AF* value to the maximum *RA* value. This crack classification method produces results that are less dependent on the type of sensor used. Figure 7 shows the *RA*-*AF* relationship and the statistical results of tensile–shear cracks under the influence of different activator moduli and water–cement ratios.

When the modulus is low (0.8 and 1.0), the proportion of tensile cracks is higher (66.71% and 67.94%), indicating that the geopolymer sample is mainly damaged by tensile damage under the action of external forces, the cracks expand more uniformly, and the fracture mode is relatively stable. As the modulus increases to 1.2, the proportion of shear cracks rises to 38.05%. In addition, at a low water–cement ratio (0.42), the crack propagation of the geopolymer sample is mainly tensile cracks (accounting for 65.46%). As the water–cement ratio increases (0.43 and 0.44), the proportion of tensile cracks slightly decreases, and the proportion of shear cracks gradually increases, reaching 34.79% and 38.05%, respectively. When the water–binder ratio is 0.45, the proportion of shear cracks increases significantly, reaching 44.19%, indicating that the crack propagation process gradually changes to shear failure.

Based on the macroscopic failure patterns of the samples, it was observed that at lower activator moduli (0.8 and 1.0), the dominant failure mode was tensile failure. However, as the activator modulus increased to 1.2, a combined tensile–shear failure mode was observed. When the water-to-binder ratio was 0.42, the samples exhibited a tensile failure mode. As the ratio increased from 0.43 to 0.45, the failure mode transitioned to a combined tensile–shear type.

In summary, the changes in the activator modulus and water–binder ratio significantly affect the crack propagation within geopolymer samples; there is a tendency of tensile failure to shear failure, ultimately leading to a failure mechanism where tensile and shear cracks interact competitively. Overall, the number of tensile cracks consistently exceeds that of shear cracks, which is consistent with the findings of Ouyang et al. [29] on cement–fly ash materials for underground structures. This is mainly attributed to the brittle nature of the material. Such inorganic cementitious materials generally exhibit low tensile strength and high compressive strength, making them more prone to tensile failure along the direction of maximum principal tensile stress under external loading. Therefore, both cement–fly ash systems and geopolymer systems typically exhibit failure modes dominated by tensile cracking.

## 4. Energy Evolution Law and Damage Constitutive Model

### 4.1. Energy Evolution Law

During the loading process of the specimen, energy is continuously input, stored, and dissipated. Assuming no heat exchange with the external environment in this physical process, the following relationship exists according to the first law of thermodynamics [40]:

(5) $$U=U_{e}+U_{d}$$

(6) $$U_{e}=\frac{1}{2E_{u}}\left[\sigma_{1}^{2}+\sigma_{2}^{2}+\sigma_{3}^{2}-2\bar{\mu }\left(\sigma_{1}\sigma_{2}+\sigma_{2}\sigma_{3}+\sigma_{1}\sigma_{3}\right)\right]$$

where *U* represents the total work input by external forces, *U_d_* the dissipated strain energy of the element, and *U_e_* the releasable elastic strain energy of the element. *E_u_* and $\bar{u}$ denote the unloading elastic modulus and unloading Poisson’s ratio of the specimen, respectively, while *σ* and *ε* denote the stress and strain of the specimen, respectively. In calculating the releasable elastic strain energy, the initial elastic modulus *E*_0_ is used instead of the unloading elastic modulus *E_u_*.

During the uniaxial compression process, only the axial stress performs work; therefore, the strain energy of each part of the filling body per unit volume can be expressed as follows [41]:

(7) $$U=\int_{0}^{\varepsilon_{1}}\sigma_{1}d\varepsilon_{1}$$

(8) $$U_{e}=\frac{1}{2}\sigma_{1}\varepsilon_{e}=\frac{1}{2E_{0}}\sigma_{1}^{2}$$

(9) $$U_{d}=U-U_{e}$$

**Figure 8:**
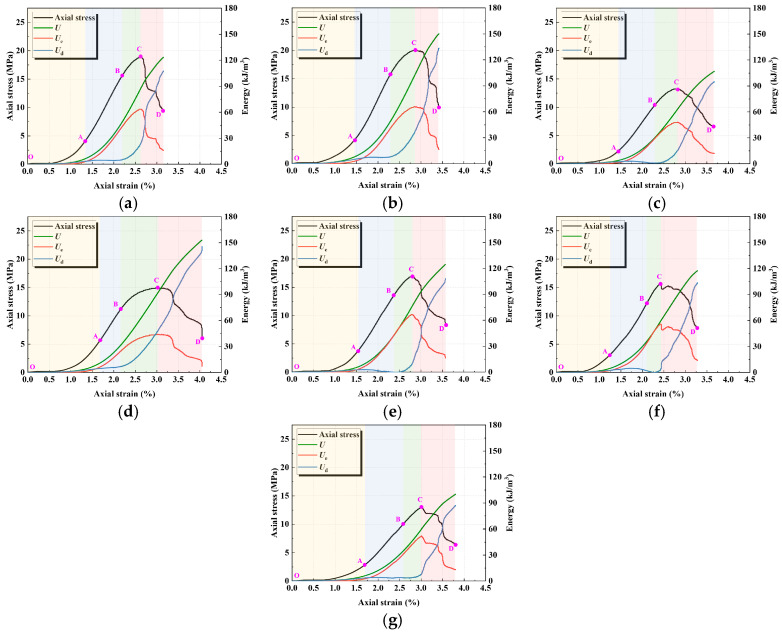
Energy evolution law: (**a**) M0.8; (**b**) M1.0; (**c**) M1.2/WB0.44; (**d**) M1.4; (**e**) WB0.42; (**f**) WB0.43; (**g**) WB0.45.

The energy evolution of geopolymer under different activator moduli and water-to-binder ratios is shown in Figure 8. It can be observed that, from the perspective of the overall energy evolution path, *U* gradually accumulates with increasing load; prior to the peak (OC stage), *U_e_* continuously increases while *U_d_* rises steadily; after the peak (CD stage), *U_e_* is rapidly released and decreases, whereas the growth rate of *U_d_* increases significantly.

At lower modulus (Figure 8a,b), *U_d_* rises rapidly in the CD stage, accompanied by a sharp drop in *U_e_*, exhibiting a brittle energy evolution characteristic of rapid release. With increasing modulus (Figure 8c,d), the rising slopes of *U*, *U_e_*, and *U_d_* gradually decrease. In particular, during the post-peak stage, the descending trend of *U_e_* and the ascending trend of *U_d_* become less pronounced, indicating a more stable energy dissipation process and thereby reflecting reduced material brittleness.

A lower water-to-binder ratio (Figure 8e) leads to a denser matrix, resulting in the rapid pre-peak (OC stage) accumulation of *U* and *U_e_*; however, in the post-peak stage (CD), *U_e_* drops sharply while *U_d_* increases rapidly. With increasing water-to-binder ratio, particularly at 0.45 (Figure 8g), the pre-peak accumulation of *U* and *U_e_* becomes slower, and in the post-peak stage, *U_e_* exhibits a gradual, stepwise decline. This is attributed to the increase in porosity induced by a higher water-to-binder ratio, where the pore effect weakens the accumulation capacity of *U_e_*, and the energy transfer among *U*, *U_e_*, and *U_d_* becomes smoother, corresponding to reduced material brittleness.

### 4.2. Damage Constitutive Model

Before establishing the damage constitutive model of geopolymer materials, the following fundamental assumptions are proposed to reasonably describe their mechanical response under uniaxial compression and to simplify the theoretical derivation:

(1) The material is assumed to be homogeneous and isotropic before loading. During loading, the occurrence of damage leads to stiffness degradation but does not induce macroscopic anisotropy within the elastic stage.

(2) The deformation of the specimen satisfies the small strain assumption, and the strain field remains continuous throughout the loading process.

(3) The damage variable (D) is defined as a scalar that reflects the degree of degradation in the material’s overall mechanical performance. Its value increases monotonically with plastic deformation and energy dissipation.

(4) The stress–strain relationship of the material follows the strain equivalence principle, meaning the constitutive relation of the damaged material can be obtained by introducing an effective stress into the stress expression of the undamaged material.

(5) During the loading process, the total energy absorbed by the material consists of recoverable elastic strain energy and unrecoverable dissipated strain energy. The former corresponds to elastic recovery, while the latter is associated with the propagation of internal microcracks and irreversible deformation.

During the test, the elastic strain energy primarily drives the elastic deformation of the specimen and is recovered with its release, while the dissipated strain energy mainly contributes to internal damage and plastic deformation. To quantitatively investigate the damage evolution during the loading process, the ratios of elastic strain energy and dissipated strain energy to the total absorbed energy at any loading state are defined as the elastic strain energy ratio (*P_e_*) and the dissipated strain energy ratio (*P_d_*), respectively, which reflect the integrity and the damage degree of the specimen [42].

According to the relationship between strain energy evolution and the development of specimen damage, *P_d_* is employed to characterize the damage variable as follows:

(10) $$D=1-\exp(-P_{d}/P_{a})$$

where *P_a_* denotes the average value of *P_d_*.

According to continuum damage mechanics, the damage constitutive model can be expressed as outlined below:

(11) $$\sigma_{c}=E_{0}\varepsilon_{1}(1-D)=E_{0}\varepsilon_{1}\exp(-P_{d}/P_{a})$$

where *σ_c_* denotes the stress calculated by the model.

**Figure 9:**
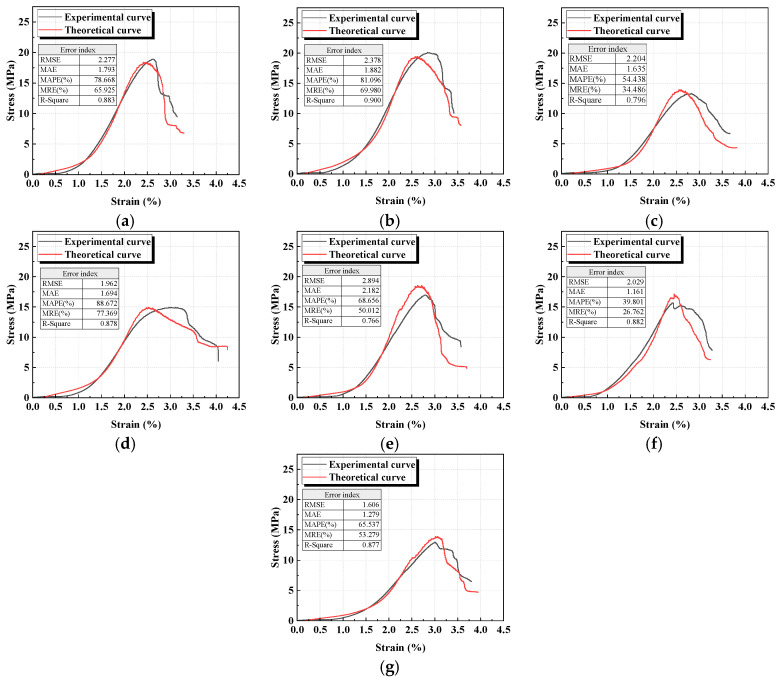
Comparison between experimental and theoretical curves: (**a**) M0.8; (**b**) M1.0; (**c**) M1.2/WB0.44; (**d**) M1.4; (**e**) WB0.42; (**f**) WB0.43; (**g**) WB0.45.

The comparison between the experimental and theoretical curves of geopolymer under different activator moduli and water-to-binder ratios is shown in Figure 9. The fitted parameters are shown in the figure. The results indicate that the theoretical curves are in strong agreement with the experimental ones, effectively representing the stress–strain curves of geopolymer specimens with varying activator moduli and water-to-binder ratios. This further provides a useful reference for the engineering design of geopolymers.

In engineering practice, when used as a supporting material in underground spaces, the constitutive model of geopolymer is primarily applied to simulate its mechanical response and failure mechanisms under complex stress conditions. By embedding the constitutive model into finite element or numerical simulation software (such as ABAQUS 2021, FLAC^3D^ 5.0, etc.), it can be used to analyze the load-bearing capacity, crack evolution, and failure modes of underground structures such as geopolymer backfills under confining pressure and mining-induced disturbances. The implementation of the model involves steps such as parameter calibration, structural modeling, load application, and numerical computation, ultimately providing theoretical support and predictive basis for the design optimization and stability evaluation of support structures.

## 5. Conclusions

The effects of different activator moduli (0.8, 1.0, 1.2, and 1.4) and water–binder ratios (0.42, 0.43, 0.44, and 0.45) on the mechanical properties and damage mechanisms of lithium-slag-based geopolymer were investigated by uniaxial compression and AE monitoring. The conclusions are drawn as follows:

1. The elastic modulus, peak stress, crack closure stress, and plastic deformation stress show an increase–decrease–increase trend with the increase in activator modulus, while they gradually decrease with the increase in water–binder ratio. The variation trend in peak strain is opposite to the above variation trend. Considering various stress indicators and elastic modulus, the optimal activator modulus is 1.0 and the optimal water–binder ratio is 0.42 for the prepared geopolymer.
2. When the modulus (0.8 and 1.0) and water–binder ratio (0.42) are lower, AE events such as the ringing count, *b*-value, and *S*-value are relatively stable, indicating that the generation and propagation of cracks inside the geopolymer are relatively slow. With the increase in modulus (1.2 and 1.4) and water–binder ratio (0.43–0.45), AE events increase significantly, indicating more frequent crack propagation and stress mutation inside the geopolymer. Additionally, the brittle failure characteristics of the geopolymer are more obvious.
3. At a lower modulus (0.8 and 1.0) and a lower water–binder ratio (0.42), the proportion of tensile cracks is high, the crack propagation is relatively uniform, the fracture mode is relatively stable, and the sample presents a macroscopic tensile failure mode. With the increase in modulus and water–binder ratio, the proportion of shear cracks gradually increases (but the failure of geopolymer is still dominated by tensile cracks), and the sample presents a tensile–shear composite failure mode.
4. At lower activator modulus (0.8 and 1.0), the energy evolution exhibits a brittle characteristic of rapid release. With increasing modulus, the growth slopes of *U*, *U_e_*, and *U_d_* progressively decrease, indicating a more stable energy-dissipation process. A lower water-to-binder ratio (0.42) leads to rapid pre-peak accumulation of *U* and *U_e_*, whereas post-peak *U_e_* drops rapidly and *U_d_* rises rapidly. As the water-to-binder ratio increases, *U_e_* shows a slower, stepwise post-peak decline, corresponding to reduced material brittleness.

This study investigated the effects of activator modulus and water-to-binder ratio on the mechanical properties and damage mechanisms of lithium-slag-based geopolymers through uniaxial compression and acoustic emission tests. The results preliminarily indicate that geopolymers prepared with LS can be applied to mine backfilling, which would contribute to the recycling and utilization of LS. However, this study focused only on the mechanical properties of LS-based geopolymers cured for 28 days. In future work, we recommend analyzing the mechanical properties at multiple curing ages and exploring the durability and cyclic loading behavior of the material for underground structural applications.

## Figures and Tables

**Table 1:** Chemical composition of LS, FA, and SG (wt%).

Raw Material	SiO_2_	Al_2_O_3_	Fe_2_O_3_	CaO	MgO	K_2_O	SO_3_	Others
LS	49.22	18.22	2.72	10.66	0.58	0.90	15.30	2.40
FA	42.80	28.60	2.62	18.35	0.93	1.05	3.22	2.43
SG	34.50	17.70	1.03	34.00	6.01	0.42	1.64	4.70

**Table 2:** The mixture proportions of the geopolymer.

Specimen	Modulus of Activator	Water-to-Binder Ratio	LS (wt%)	FA (wt%)	SG (wt%)	Activator (wt%)	Sodium Silicate (g)	NaOH (g)
M0.8	0.8	0.44	9	41	50	10	78	37
M1.0	1.0	0.44					89	26
M1.2/WB0.44	1.2	0.44					97	18
M1.4	1.4	0.44					102	13
WB0.42	1.2	0.42					97	18
WB0.43	1.2	0.43					97	18
WB0.45	1.2	0.45					97	18

## Data Availability

All data, models, and code generated or used during this study appear in the published article.
